# Dynamic assembly, localization and proteolysis of the *Bacillus subtilis *SMC complex

**DOI:** 10.1186/1471-2121-6-28

**Published:** 2005-06-29

**Authors:** Judita Mascarenhas, Arsen V Volkov, Cornelia Rinn, Jens Schiener, Reinhard Guckenberger, Peter L Graumann

**Affiliations:** 1 Biochemie, Fachbereich Chemie, Hans-Meerwein-Straße, Philipps-Universität Marburg, 35032 Marburg; 2 Institut für Zytobiologie und Zytopathologie, Robert-Koch Str. 6, 35037 Marburg, Germany; 3 The Scripps Research Institute, Department of Molecular and Experimental Medicine, La Jolla, CA92037, USA; 4 Max-Planck-Institut für Biochemie, Abteilung Molekulare Strukturbiologie, Am Klopferspitz 18, 82152 Martinsried, Germany; 5 Institut für Mikrobiologie, Verfügungsgebäude, Stefan-Meier Str. 19, Universität Freiburg, 79104 Freiburg, Germany

## Abstract

**Background:**

SMC proteins are key components of several protein complexes that perform vital tasks in different chromosome dynamics. Bacterial SMC forms a complex with ScpA and ScpB that is essential for chromosome arrangement and segregation. The complex localizes to discrete centres on the nucleoids that during most of the time of the cell cycle localize in a bipolar manner. The complex binds to DNA and condenses DNA in an as yet unknown manner.

**Results:**

We show that *in vitro*, ScpA and ScpB form different complexes with each other, among which the level of the putative 2 ScpA/4 ScpB complex showed a pronounced decrease in level upon addition of SMC protein. Different mutations of the ATPase-binding pocket of SMC reduced, but did not abolish interaction of mutant SMC with ScpA and ScpB. The loss of SMC ATPase activity led to a loss of function *in vivo*, and abolished proper localization of the SMC complex. The formation of bipolar SMC centres was also lost after repression of gyrase activity, and was abnormal during inhibition of replication, resulting in single central clusters. Resumption of replication quickly re-established bipolar SMC centres, showing that proper localization depends on ongoing replication. We also found that the SMC protein is subject to induced proteolysis, most strikingly as cells enter stationary phase, which is partly achieved by ClpX and LonA proteases. Atomic force microscopy revealed the existence of high order rosette-like SMC structures *in vitro*, which might explain the formation of the SMC centres *in vivo*.

**Conclusion:**

Our data suggest that a ScpA/ScpB sub-complex is directly recruited into the SMC complex. This process does not require SMC ATPase activity, which, however, appears to facilitate loading of ScpA and ScpB. Thus, the activity of SMC could be regulated through binding and release of ScpA and ScpB, which has been shown to affect SMC ATPase activity. The proper bipolar localization of the SMC complex depends on a variety of physiological aspects: ongoing replication, ATPase activity and chromosome supercoiling. Because the cellular concentration of SMC protein is also regulated at the posttranscriptional level, the activity of SMC is apparently regulated at multiple levels.

## Background

SMC-like proteins are a widespread protein family performing essential tasks in a variety of chromosome dynamics [1]. True SMC proteins are invariably associated with additional proteins and form part of at least 3 different complexes in lower and 4 complexes in higher eukaryotes: SMC 1 and SMC 3 form at part of the cohesin complex that bridges sister chromosomes during S phase, ensuring proper alignment of chromosomes during metaphase [2]. SMC 2 and SMC 4 form the condensin complex, which mediates chromosome structure and condensation throughout the cell cycle and most significantly during prophase [3]. Both, cohesin and condensin also have subunits belonging to the kleisin protein family that share similar N – and C-termini, and one or two other non-SMC subunits. Higher eukaryotes contain at least 2 condensin complexes, in which SMC 2/4 interact with different non SMC proteins, and which perform different structural tasks in the establishment of chromosome structure [4]. SMC 5 and 6 are part of a large DNA repair complex that contains at least 7 additional proteins [5]. Additionally, SMC proteins are present in the dosage compensation complex that mediates transcriptional repression in *C. elegans *[6]. In prokaryotes, only one true SMC protein is present in most genomes thus far analysed, which forms a complex with ScpA, a kleisin protein, and ScpB [7-9]. The prokaryotic SMC complex is required for proper chromosome arrangement and for active segregation of sister chromosomes into opposite cell halves before division [10-12].

Thus, SMC proteins act in a wide variety of different protein complexes, and it is an important question how this protein family achieves these different functions at a molecular level. SMC proteins consist of an N-terminal region containing the nucleotide binding Walker A motif, one part of the ATP binding pocket, two long central coiled coil regions separated by a hinge domain, and a C-terminal region that contains a Walker B motif (the other part of the ATP binding pocket) and a "C"-motif required for ATP hydrolysis [13]. The coiled coil regions fold back onto each other, forming a 50 nm coiled coil. The functional ATPase pocket is jointly formed by the N-and the C-terminal regions, which form the head domain, as revealed in the crystal structures [14,15]. SMC proteins generally form dimers [16,17], mediated by the hinge domain [18]. So, SMC proteins generally form symmetrical dimers, composed of a central hinge and two long arms with two head domains at their ends. These molecules can adopt an open V-shape, or a closed structure, with closure apparently occurring at the head domains. SMC interactors have so far all been found to bind to the head domains.

*In vitro*, the condensin complex introduces positive writhe (a right handed super helix) into DNA [19], which probably leads to the introduction of negative supercoiling *in vivo*. The cohesin complex and prokaryotic SMC have been shown to bind to DNA as a ring like structure [9,20,21], in which DNA is most likely bound by wrapping of the long coiled coil arms around the DNA, and by closing of the ring at the head domains. DNA is condensed in a highly cooperative and repetitive manner [22], but the actual mode of condensation is yet unknown. In *Bacillus subtilis*, ScpA requires ScpB for efficient binding to the SMC head domain [9]. Both, ScpA and ScpB are needed for chromosome compaction and segregation *in vivo *[7], and have a negative effect on SMC ATPase activity *in vitro *[23]. The SMC complex is functionally similar to the *Escherichia coli *MukBEF complex (with MukB being the SMC counterpart) [24], and localizes in a cell cycle dependent manner: at the onset of the cycle, the complex is found as a single focus towards the middle of the cells. After separation of chromosome origins towards opposite cell poles, the complex separates into two foci that rapidly move into both cell halves close to origin regions [7,25]. From these defined regions on the nucleoids, the SMC complex compacts and organizes the chromosomes as they are ejected from the central polymerase complex [9,26]. How the specific localization of the complex is achieved is still unclear.

In this work, we have investigated the mode of complex formation of SMC, ScpA and ScpB *in vivo *and *in vitro*. We have found that ScpA and ScpB form several defined subcomplexes, one of which is preferentially recruited into the SMC complex. The complex requires proper supercoiling, SMC ATPase activity and ongoing replication for its proper localization, and is additionally regulated at the level of proteolysis. Using AFM, we have found that SMC can form striking rosette-like structures, which could be the basis of the subcellular centres formed in cells.

## Results

### ScpA and ScpB form different complexes in the absence of SMC

**Figure 1:**
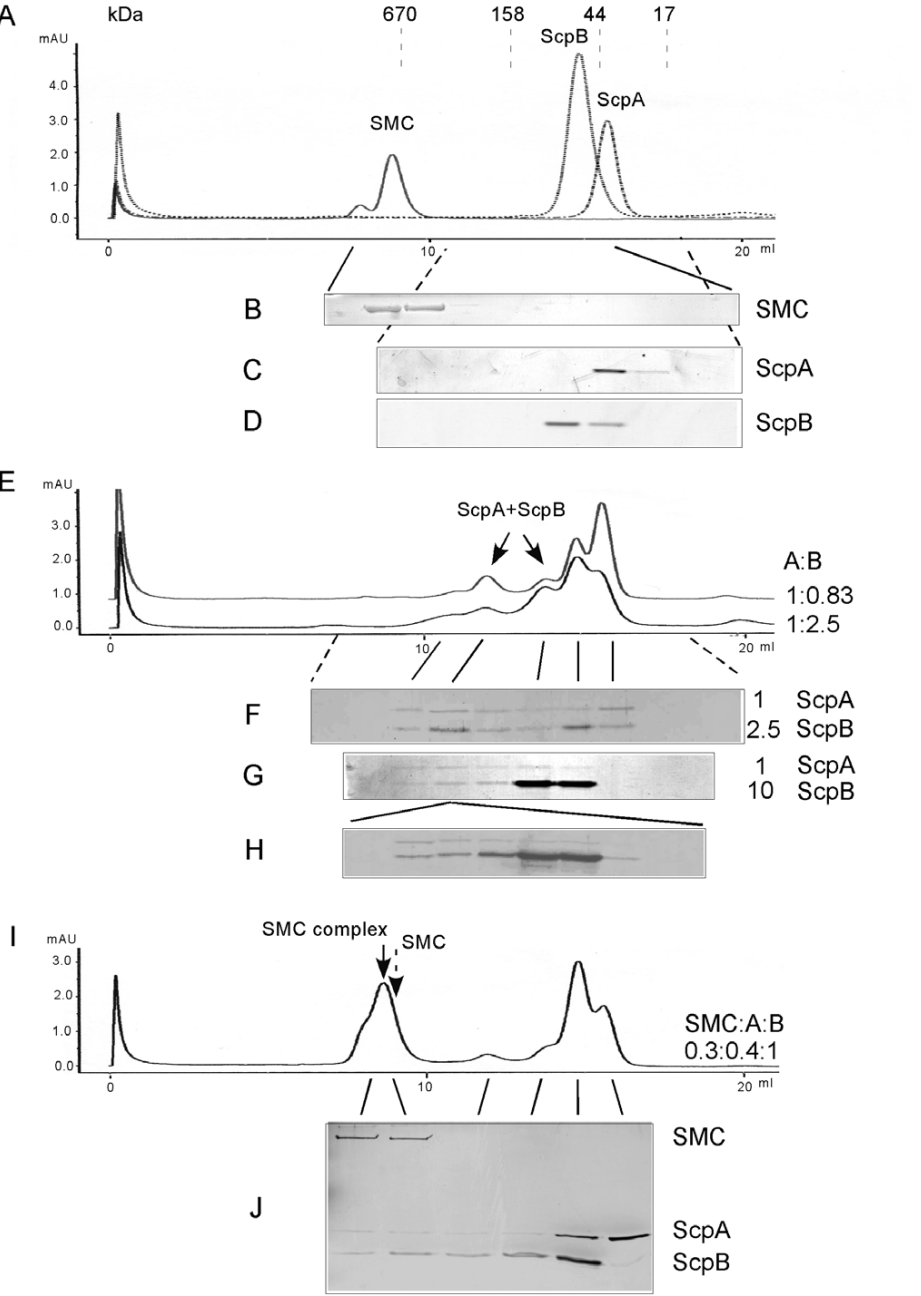
Analytical gel filtration assays with SMC and ScpA and ScpB. A) Reference runs for purified SMC (0.3 nmol), ScpA (0.4 nmol) and ScpB (1 nmol), all three profiles were merged. Dashed lines show the position of peaks for proteins of gel filtration standards. The void volume peak is indicated by a white triangle. B-D) Coomassie stained SDS PAGE showing continuous fractions collected during gel filtration. Full or dashed lines show the regions from which the fractions were taken. E) ScpA mixed with ScpB in two different proportions, 1:2.5 nmol (lower graph) and 1:0.83 nmol (upper graph). Arrows indicate the existence of at least two discrete peaks containing different forms of the ScpA/B complex. F-G) Coomassie blue-stained SDS-PAGE showing continuous fractions collected during gel filtration. Dashed lines indicate the region from which the fractions were taken, full lines indicate the position of the fractions within the elution profile, indicating peak elution of ScpA, ScpB or of the distinct ScpA/B complexes. G) corresponds to (F) with a 1:10 ration of ScpA to ScpB. H) The elution peak at 12 ml is subjected to a second round of gel filtration analysis, and fractions containing ScpA and ScpB are shown (stained with silver), I) SMC (0.3 nmol) incubated with ScpA (0.4 nmol) and ScpB (1 nmol) before gel filtration. The dashed arrow shows the position of the SMC peak in the absence of Scps, and the solid arrow peak elution of the SMC complex. J) Silver-stained SDS-PAGE with (non-continuous) fractions loaded corresponding to elution peaks from gel filtration (indicated by solid lines) shown in I). Note that the first two lanes in (J) correspond to the first two lanes in (B) and in (F), i.e. that SMC shifts to a higher molecular weight in complex with ScpA/B, and the ScpA/B are not present in these fractions in the absence of SMC (F).

ScpA and ScpB have been shown to interact with each other in the absence of SMC protein [23,27]. To investigate the nature of this interaction, we used native gel electrophoresis. Incubation of freshly purified ScpA and ScpB resulted in the formation of at least two additional bands that migrated much slower through the gel (data not shown), showing that ScpA and ScpB form at least two different stable complexes in the absence of SMC. To obtain more information on the nature of the interaction, we employed analytical gel filtration assays. ScpB forms a stable dimer in solution, with peak elution at 15.0 ml (Fig. 1A), while ScpA is mostly in a monomeric form in solution (peak elution at 16.2 ml, Fig. 1A), but a small fraction is also present in a dimeric form (the ScpA dimer is detectable using a column with higher resolution below 75 kDa, rather than in a column with a resolution range up to 1.5 mDa as used in this study) [9]. Fig. 1E shows that upon mixing of ScpA and ScpB, both peaks underwent a pronounced decrease in favour of two new peaks at 13.9 ml and at 12.0 ml (corresponding to protein masses of 110 and 210 kDa, respectively, Fig. 1E, lower profile, compare with 1A). Fig. 1F shows that both, ScpA and ScpB, were present in a substantial amount in the fractions corresponding to the new peaks, while they were undetectable in these fractions in the absence of the other Scp (Fig. 1C and 1D). Thus, ScpA and ScpB form at least two distinct stable complexes in the absence of SMC. ScpA and ScpB were also present at even higher molecular weight fractions (Fig. 1E and 1F, shoulder at 11 ml) when incubated together, indicating that ScpA and ScpB form even higher multimeric structures. These findings were confirmed with sucrose gradient centrifugation experiments, where a 1:1 mixture of both proteins showed a strong interaction, both proteins were found in considerably higher molecular weight fractions, compared to the single proteins (data not shown).

**Figure 2:**
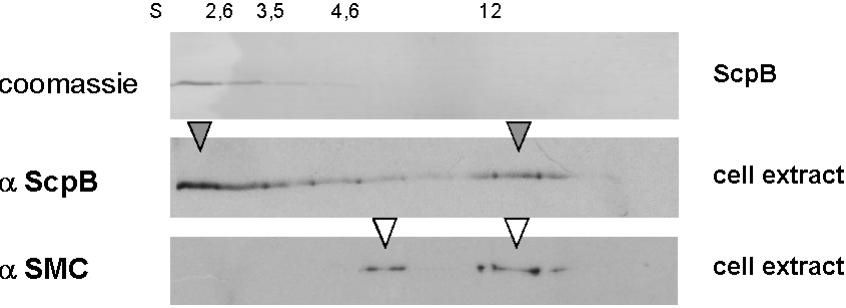
SDS PAGE showing fractions eluted after sucrose gradient centrifugation using 5 to 20% sucrose concentration. 0.4 nmol of purified ScpB (upper panel, Coomassie stained), cell extract probed with ScpB antiserum (middle panel) or with SMC antiserum (lower panel). Lower and middle panels are Western blots. Arrowheads indicate the elution peaks for ScpB (grey) or for SMC (white). Sedimentation peaks of marker proteins are shown on the top.

Because ScpA and ScpB alone elute at volumes that correspond to molecular sizes of 35 kDa and 65 kDa, respectively (Fig. 1A, 1C and 1D), the new peaks containing the ScpA/ScpB complex most likely consist of a ScpA monomer and a ScpB dimer (110 kDa peak) and two ScpA molecules (or rather a ScpA dimer) and two ScpB dimers (210 kDa peak). However, we cannot exclude that the 210 kDa peak consists of a ScpA dimer and a ScpB dimer. Increasing the relative amount of ScpA resulted in a pronounced increase of the 210 kDa peak, and in a decrease of the 110 kDa peak (Fig. 1E, upper profile). On the other hand, after an increase in the relative amount of ScpB, ScpA was exclusively found in higher molecular weight fractions, and no longer as a monomer (Fig. 1G), showing that all molecules were in complex with ScpB. Possibly, ScpB is a limiting factor for the formation of high molecular weight ScpA/ScpB complexes formed by multimers of ScpA that are favoured by the interaction with ScpB. ScpA is predicted to contain a long coiled coil region in the central region of the protein [7], which might mediate formation of dimers (a sub-fraction of ScpA forms dimers in solution) and of multimers. When the 210 kDa complex was subjected to another gel filtration run, ScpA and ScpB eluted in the same elution pattern as when freshly mixed (Fig. 1H), showing that ScpA and ScpB form a dynamic, reversible complex, dependent on the concentration of each complex partner. To test if ScpA/ScpB subcomplexes might exist *in vivo*, we performed sucrose gradient experiments. Fig. 2 shows that in cell extract, ScpB is present in two major fractions, corresponding to 1.8S and to 12-13S units (middle panel). The low molecular weight fraction corresponds to free ScpB [28], while the high molecular weight fraction corresponds to the SMC complex, since SMC is also present in this fraction (lower panel, SMC is also present at 6.5S, corresponding to unbound SMC dimer [29]). Purified ScpB is present in fractions up to about 4.6S units (Fig. 2, upper panel), while in cell extract, a considerable amount of ScpB is also present at 4.6S and above, most likely corresponding to ScpA/ScpB subcomplexes. While these experiments are no proof for ScpA/B complexes, they strongly support the idea that they exist *in vivo*.

### A ScpA/ScpB sub-complex is recruited into the SMC complex in vitro

For further investigation of interactions of SMC, ScpA and ScpB *in vitro*, and to gain more information on the composition of the complex, we performed analytical gel filtration assays. Gel filtration of SMC alone resulted in a peak elution at a size corresponding to 690 kDa (Fig. 1A and 1B). However, because of its long coiled coil regions, SMC is a highly elongated molecule, so that this size is greatly exaggerated. The more realistic molecular mass of this fraction can be calculated from data gained in sucrose gradient centrifugation: purified SMC has a sedimentation coefficient of 6.6 (data not shown, [29]), and combined with a stokes radius of 8.5 (as deduced from elution at 690 kDa), the native weight is 260 kDa, strongly suggesting that SMC is mostly present as a dimer (monomeric size 135 kDa) *in vitro*. Addition of ScpA or of ScpB alone did not result in any interaction, that is the SMC peak did not shift, and the 690 kDa fraction did not contain any ScpA or ScpB as examined by SDS-PAGE (data not shown). However, if both, ScpA and ScpB, were incubated with SMC, a considerable shift of the SMC peak to a bigger size (780 kDa) and an increase in its magnitude was apparent (Fig. 1I, indicated by solid and dashed arrows, and compare the first lanes in Fig. 1B and in 1J). Complex formation was accompanied by the decrease of the ScpA and ScpB peaks, and of the larger ScpA/ScpB complex peak (210 kDa, compare lower profile in Fig. 1E with 1I). It should be noted that the separation range of the column reaches up to 1.5 MDa, so the shift of the elution peak is well within the range of resolution. SDS PAGE confirmed the presence of ScpA and of ScpB in the high molecular weight fractions (Fig. 1J, compare with 1C, D and 1F). Addition of different ratios of SMC, ScpA or of ScpB did not considerably change the binding profile (data not shown). Interestingly, the 210 kDa peak, but not the 110 kDa peak of the ScpA/B complex showed a decrease after incubation with SMC (compare Fig. 1E, lower profile, with Fig. 1I). The 210 kDa peak showed a 2.5-fold decrease after addition of SMC protein (from 1.1 to 0.4 units), while the individual ScpA and ScpB peaks were reduced by a factor of 2 and 1.5, suggesting that the putative 2ScpA/4ScpB complex is a source of ScpA and ScpB for recruitment to the SMC complex. However, because ScpA and ScpB are in a dynamic equilibrium, it is also possible (although less likely) that the 110 kDa complex is recruited into the SMC complex.

### ATP-binding or hydrolysis are important but not required for complex formation with ScpA and ScpB

**Figure 3:**
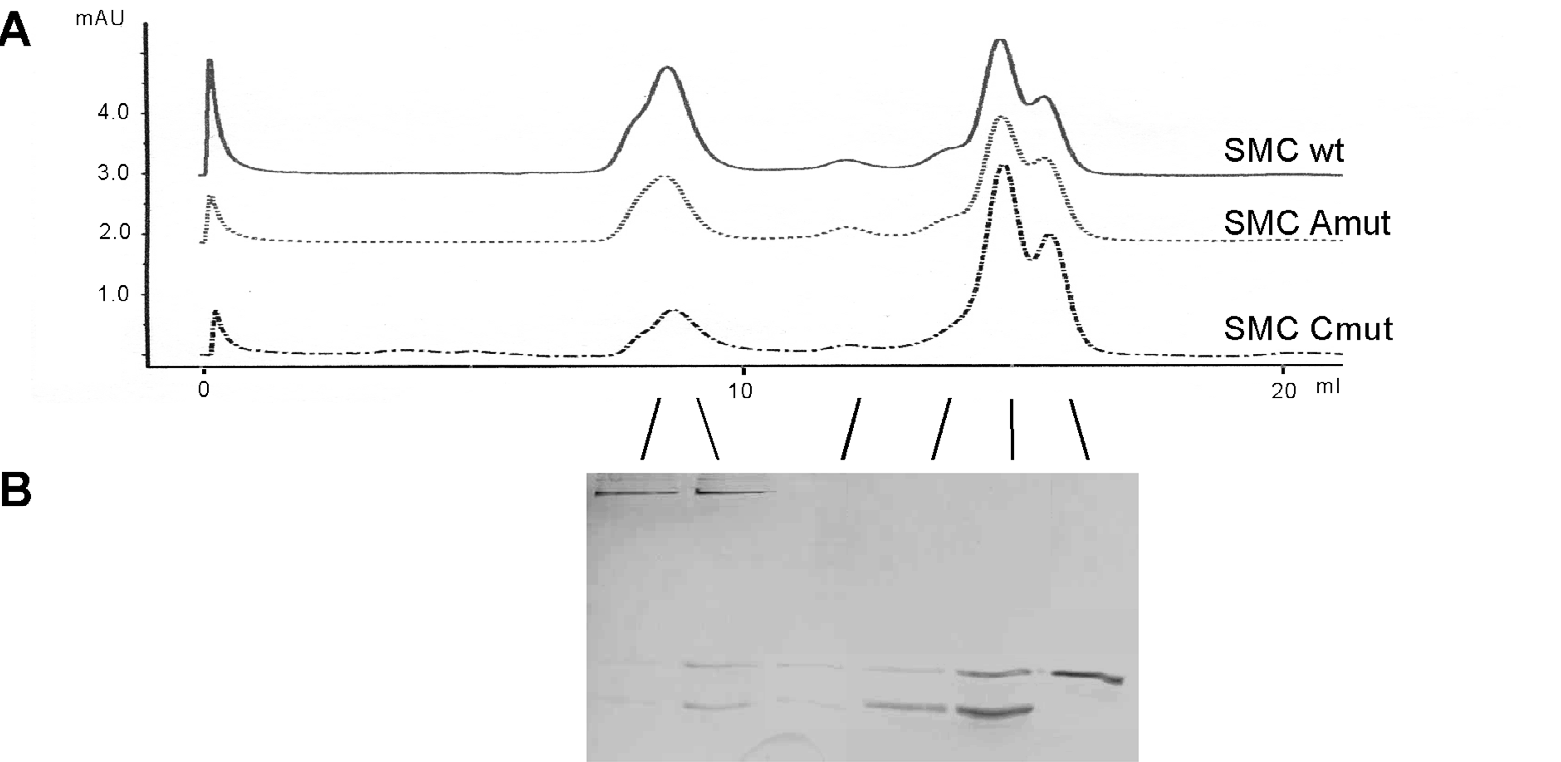
Analytical gel filtration assays with SMC and SMC mutant proteins. A) Comparison of gel filtration profiles of wild type SMC and SMC mutants (as noted on the profiles) incubated with ScpA and ScpB, same amounts as in Fig 1I). 1B) Silver stained SDS-PAGE with fractions loaded corresponding to elution peaks from gel filtration of SMC Walker A mutant incubated with ScpA and ScpB (corresponding to Fig. 1J).

The head domains (hd) of SMC proteins share three conserved motifs with the ATP-binding cassette (ABC) family of ATPases: Walker A and Walker B motifs and the so-called C-motif, also known as signature motif. Indeed, SMC possesses a weak ATPase activity *in vitro *[29]. To study the importance for complex formation of the ATPase activity in SMC, we introduced two point mutations into SMC. A K37I substitution in the Walker A motif abolishes ATP binding activity [21], while a S1090R substitution in the C-motif allows for ATP binding but prevents ATP hydrolysis [21]. Thus, these mutations can distinguish between the two stages of ATPase activity in SMC (which overall is very low, [29]). Gel filtration experiments with wild-type SMC protein and with both mutant forms showed that each mutant protein was still able to form a complex with ScpA and ScpB, that is, all SMC peaks increased and shifted to the same elution peak when mixed with ScpA and ScpB (Fig. 3A). However, the increase in the SMC complex peak at 750 kDa was lower for the Walker A mutant, and even more so for the C-motif mutant protein compared to the wild-type protein (Fig. 3A and 3B). Fractions corresponding to the SMC complex contained ScpA and ScpB for wild type and both SMC mutants, but the amounts of ScpA and of ScpB in the mutant SMC complexes were considerably lower compared to wild-type SMC complex (Fig. 3A, peaks for A-mutant or C-mutant complexes are 35% or 50% reduced compared to wild-type SMC, with the same amounts of SMC or mutant proteins present in these fractions, and Fig. 3B, compare with 1J). The presence or absence of ATP in the binding reactions did not change the results (data not shown). Thus, we conclude that ATP-binding and hydrolysis are not required for SMC complex formation, but that both mutations affect the efficiency of the interaction. Unfortunately, we were not able to conclusively investigate the effect of Walker A and C motif mutations on the DNA-binding activity of SMC.

### SMC variants carrying mutations in the Walker A and C ATPase motifs are non-functional and cannot mediate proper subcellular localization of the SMC complex in vivo

**Figure 4:**
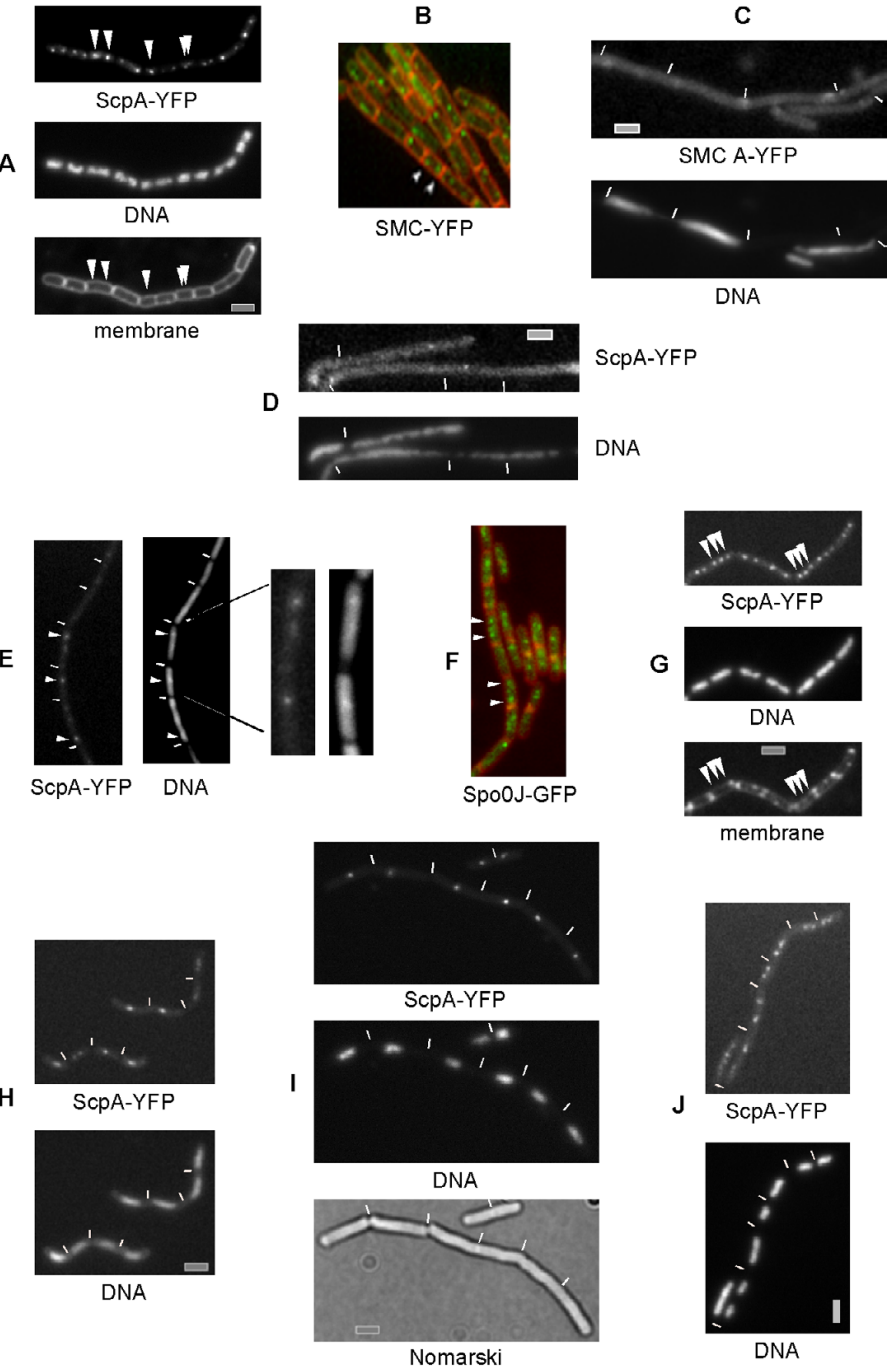
Localization of the SMC complex in live cells depends on several physiological factors. A) Localization of ScpA-YFP in wild-type cells (arrowheads indicate central SMC foci in a newborn cell, two central foci in a slightly older cell, and bipolar foci in a yet older cell), and B) SMC-YFP in wild type cells. C) defective localization of the Walker A motif SMC-YFP mutant protein, D) localization of ScpA-YFP in a strain expressing the C-motif SMC mutant allele only, E) localization of ScpA-YFP, 1 hour after addition of nalidixic acid to block gyrase activity (arrowheads indicate remaining foci). F) localization of Spo0J-GFP, 1 hour after addition of nalidixic acid. G-J) ScpA-YFP in a *dnaB *ts strain, G) 20–30 min, H) 30–45 min or I) 60 min after shift to 42°C, J) 60 min after shift back from 42°C to 25°C. Arrowheads indicate two bipolar and one central ScpA-YFP foci in (G). White lines indicate septa between cells. Grey bars 2 μm.

The SMC complex has a specific cell cycle dependent subcellular localization [7,30]. Fig. 4A and 4B shows that in small cells, one focus localizes to the middle of the cell (single arrowhead), while in slightly larger cells, two foci are present close to the centre (two adjacent arrowheads). Still at an early time point during the cell cycle, the foci move towards opposite cell poles (two separate arrowheads, note that this is still a small cell), such that predominantly, two foci are present within each cell half. It is unclear which factors mediate this specific pattern of localization.

To investigate if Walker A or C-motif activities are important for SMC function *in vivo*, we generated two mutations, one in the Walker A motif, and one in the C motif. The mutant genes (SMC-A or SMC-C) were integrated at an ectopic site on the chromosome (*amy *locus). Expression of each mutant form did not show any effect on growth or nucleoid morphology (table 2), showing that the mutations are recessive. However, deletion of the original *smc *gene in both strains resulted in a *smc *null phenotype, i.e. temperature sensitive, slow growth below 23°C (table 2), even when the mutant proteins were overproduced (0.1 mM IPTG result in wild type SMC levels, and 1 mM IPTG to about 8 fold overproduction). In the absence of the original *smc *gene, cells were only able to grow at 37°C (or at 30°C) in the presence of the wild-type *smc *gene at the ectopic site (table 2), but not in the presence of either mutant allele, showing that both, ATP-binding and ATP hydrolysis are required for the function of SMC. When the SMC mutant alleles were fused to GFP and were expressed in the presence or in the absence of wild-type SMC protein, both fusions mislocalized, they were dispersed throughout the cells, and less than 1% of the cells contained discrete foci (Fig. 4C, and data not shown). Likewise, ScpA-YFP no longer formed bipolar foci in cells solely expressing either SMC mutant protein, but localized throughout the cells, with only 5% of the cells containing single fluorescent foci (Fig. 4D). These findings show that the proper localization of the SMC complex depends on the ATPase activity of SMC.

**Table 2:** growth of different strains

Strain	growth at 37°C	growth at 23°C	anucleate cells 23°C
PY79	+	+	-
PGΔ388 (Δ*smc*)	-	+	17%
JM51 (*smc-A *at *amy*)	+	+	-
JM52 (*smc*-*C *at *amy*)	+	+	-
JM59 (Δ*smc*, *smc-A *at amy)	-	+	14%
JM60 (Δ*smc*, *smc-C *at *amy*)	-	+	16%
EP58 (Δ*smc*, *smc *at *amy*)	+	+	-

### The formation of discrete subcellular SMC centres requires proper supercoiling

Previous studies have suggested that the SMC complex functions in concert with topoisomerases in chromosome compaction [31,32]. We wished to investigate the requirement for proper localization of the SMC complex in the context of DNA topology, so JM25 (*smc-yfp*) and JM8 (*scpA-yfp*) cells were treated with drugs that inhibited gyrase activity, since gyrase is the major enzyme generating overall negative supercoiling in the cell. When JM8 and JM25 cells were treated with novobiocin (10 μg/ml) or nalidixic acid (200 ng/ml) for one hour, 95% of the cells showed highly decondensed nucleoids. The SMC/ScpA proteins were seen as foci in only 18% of the cells, with >80% of these having a single focus (Fig. 4E), in contrast to >95% of wild type cells, 80% of which have bipolar SMC foci. Additionally, SMC or ScpA foci were much fainter compared to non-treated cells, and background fluorescence was higher throughout the cells, compared with non-treated cells (SMC protein levels were similar to non-treated cells, data not shown). Interestingly, in those cells containing SMC or ScpA foci, DNA staining showed more condensed DNA at the positions of the remaining SMC centres (see blow up of Fig. 4E), suggesting that a higher degree of DNA compaction is retained within or around the condensation centres, in spite of the loss of overall negative supercoiling. As a control, we treated cells expressing Spo0J-GFP in a similar manner. Spo0J binds to several regions close to the origin region on the chromosome, and thus is generally present as two bipolar foci in exponentially growing cells [33]. 1 hour after addition of novobiocin, 45% of the cells retained bipolar Spo0J-GFP foci (Fig. 4F), as opposed to 92% of the non-treated cells, showing that the localization of Spo0J is only moderately affected by inhibition of gyrase. These experiments show that the SMC complex requires proper supercoiling for its assembly into bipolar condensation centres. In cells still containing SMC foci after inhibition of gyrase, foci were frequently seen close to the cell centre (Fig. 4E), where the replication machinery is usually located [26]. Inhibition of gyrase increases the local accumulation of positive supercoils ahead of the replication forks and this effect strongly reduces replication fork progression [34].

### Bipolar localization of SMC centres depends on ongoing DNA replication

The possible connection between localization of the SMC complex and DNA replication prompted us to visualize the dynamic bipolar movement of the SMC complex foci in cells, in which replication can be reversibly arrested. A previous study has shown that SMC-GFP foci persist during arrest of replication [30]. To investigate the positioning of SMC foci, SMC-YFP and ScpA-YFP fusions were moved into two different strains, which carry a temperature sensitive mutation in *dnaA *or *dnaB *that block initiation of replication upon increase in temperature to 42°C. JM46 (*scpA-yfp, dnaB*^*ts*^) and JM57 (*smc-yfp, dnaB*^*ts*^) cells were monitored for ScpA or SMC localization by shifting the growing cultures from 25°C to 42°C for an hour. Already after 20–30 min, the pattern of localization changed, 40–50% of the cells contained an additional central ScpA or SMC focus (and thus three foci, indicated by arrowheads in Fig. 4G). 30 minutes and one hour after temperature upshift, all ongoing replication processes were completed but no new round was initiated. Under this condition, cells were highly elongated and filamentous, and showed a single compact nucleoid. One, and less frequently (in less than 10% of the cells) up to three ScpA foci were seen close to the cell centre on the nucleoids (Fig. 4H, 4I). A shift of wild type cells from 25 to 42°C did not markedly affect the bipolar localization of SMC or of ScpA, 15 to 20% of the cells contained 4 well separated foci rather than 2, due to an increase in growth rate (data not shown, similar to incubation of cells at 25°C in rich medium [7]). When the cultures were shifted back from 42°C to 25°C and were incubated for an additional hour, cells resumed the replication process, which was visible through the presence of two nucleoids in many cells (Fig. 4J). The cells were no longer filamentous and ScpA was seen as separated bipolar foci in >80% of the cells (Fig. 4J). SMC-YFP localized as a central focus in replication-arrested cells, and rapidly regained bipolar positioning after resumption of replication, in a manner indistinguishable from ScpA (data not shown). Similar results were obtained using a temperature sensitive *dnaA *strain (data not shown). These experiments show that bipolar localization of the SMC complex depends on active replication, and suggest that the SMC complex might be initially loaded at the replication machinery close to the cell centre, and that active chromosome segregation (which depends on replication) drives the formation of bipolar SMC centres.

### The cellular level of SMC is regulated at the posttranscriptional level

**Figure 5:**
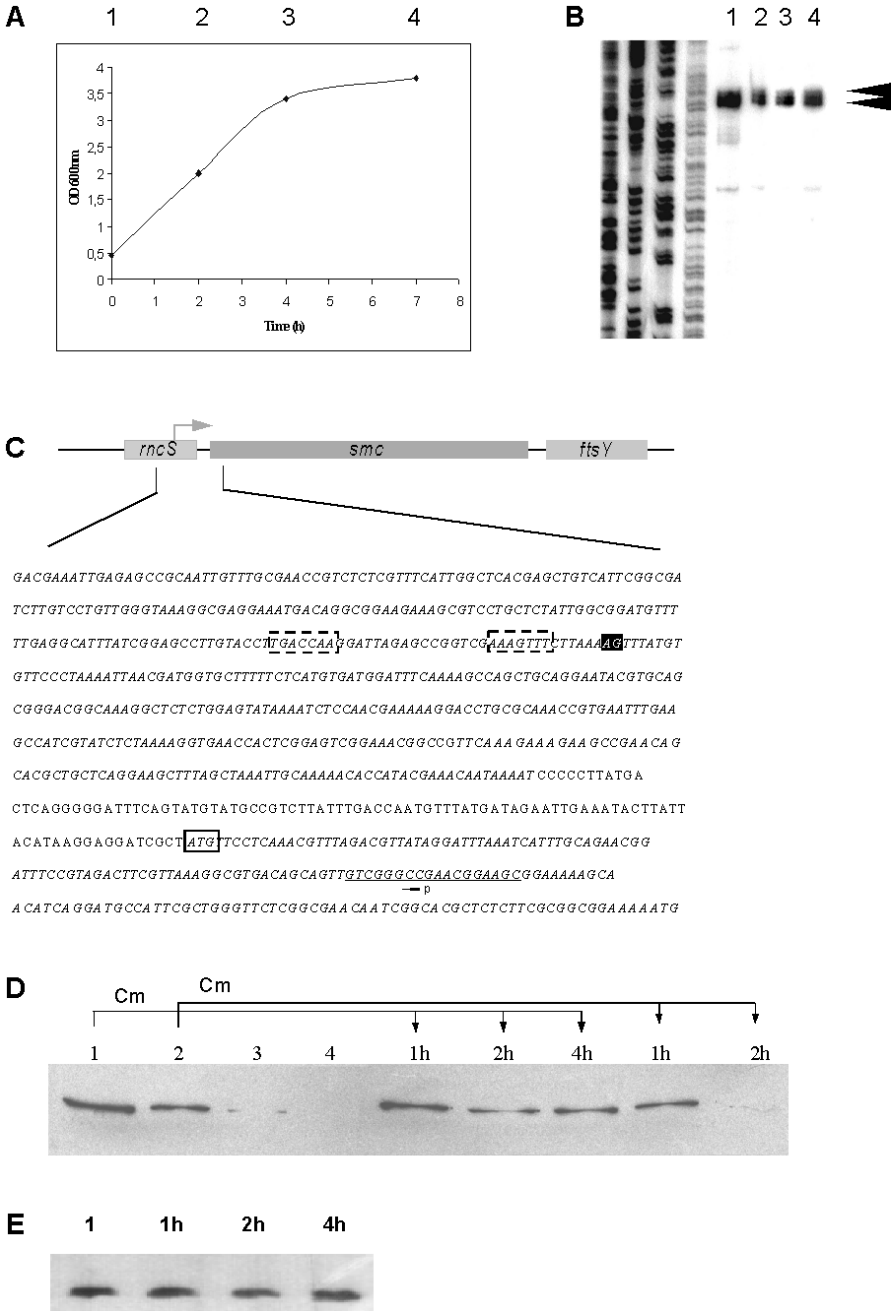
*Smc *transcription and SMC levels during different phases of growth. A) Samples 1 to 4 were taken at the time points indicated in the growth curve (note that an OD of 0.5 is in the middle of exponential growth). B) Primer extension analysis of transcripts from growth phases shown in (A), arrowheads indicate the two start bases. C) Organization of the *smc *region. The two start bases are shown in the black box. The promoter region as derived from primer extension analysis lies within the *rncS *gene as indicated, and is highlighted in the dashed boxes in the sequence. The start of the *smc *gene is boxed, and the position of the primer used is underlined. *RncS *and *smc *genes are shown in italics. D) Western blot analysis of SMC during growth phases 1 to 4 (lanes 1, 2, 3, and 4). Cells from phases 1 and 2 were incubated after addition of chloramphenicol (Cm) as indicated by the arrows for 1, 2 or 4 hours (1 h, 2 h or 4 h). E) Western blot of CspB during growth phase 1, and 1, 2 or 4 hours after addition of Cm.

Previously, SMC protein has been shown to be rapidly depleted as cells enter stationary phase [35]. To analyse if the *smc *gene is transcriptionally regulated, samples from cultures were collected in mid or late exponential phase (points 1 and 2, Fig. 5A), and in early or late stationary phase (points 3 and 4, Fig. 5A). Primer extension analyses were performed with cell extracts containing the same number of cells from these different growth phases. Fig. 5B shows a strong signal (indicated by arrowheads) that indicates the existence of a major promoter within the *rncS *gene (Fig. 5C). Clearly, mRNA levels remain relatively stable between exponential phase and stationary phase (Fig. 5B). In spite of the continued transcription of *smc*, protein levels decrease strongly from mid exponential phase to early stationary phase, and SMC becomes undetectable in stationary phase (Fig. 5D, lanes 1–4). These experiments show that synthesis of SMC is post-transcriptionally regulated. To test if SMC is proteolytically degraded at the onset of stationary phase, we arrested translation at growth points 1 and 2 by adding chloramphenicol (Cm) to the cultures to block *de novo *protein synthesis. Fig. 5D shows that after addition of Cm at growth point 1, SMC levels somewhat decrease, but remain well detectable for at least 4 hours (indicated by arrows). It should be noted that >90% of all proteins are stable in exponentially growing cells [36], and the amount of CspB protein, which we used as a control for this experiment, remained constant after addition of Cm (Fig. 5E), as well as during entry into stationary phase (data not shown). However, 2 hours after addition of Cm to late exponentially growing cells, SMC was strongly degraded (Fig. 5D, last lane). These experiments show that while SMC is partially proteolysed in growing cells, and thus during the cell cycle, it becomes highly unstable as cells enter stationary phase.

**Figure 6:**
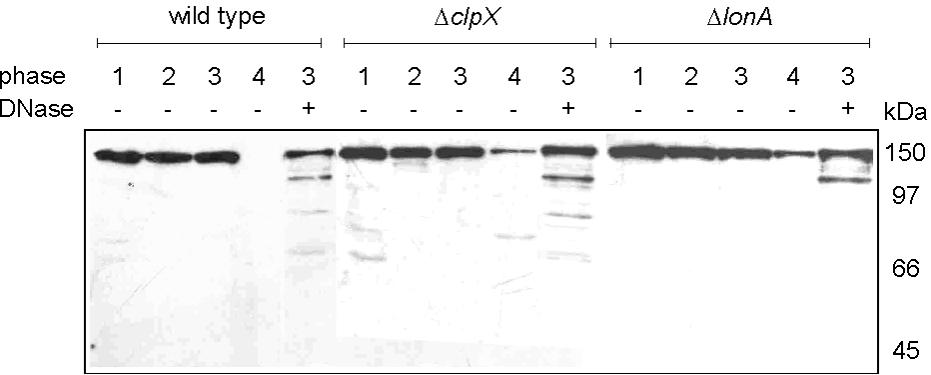
Proteolysis of SMC at the onset of stationary phase in protease-deficient strains. Lanes "1–4": levels of SMC protein in wild type, *clpX *or *lonA *mutant cells during phases 1–4 of growth as indicated in Fig. 5, lane "3+" levels of SMC protein in cell extract from phase 3 to which DNase has been added. Numbers indicate the positions of marker proteins.

To investigate which cellular proteases might be responsible for the degradation of SMC, we analysed SMC levels at the different growth points in two different protease-deficient strains. Fig. 6 shows that in the absence of ClpX, one of the factors that forms a protease complex with ClpP [37], or of LonA, another major protease [38], SMC was still detectable in cells from stationary phase, in contrast to wild-type cells (compare lanes labelled "4"). These experiments show that ClpX and LonA have an important contribution towards proteolysis of SMC. Interestingly, when DNase was added for 30 min to the cell extracts from time point "3", SMC became highly unstable, degradation products at the expense of full length SMC became well visible (Fig. 6, lane 3 "+ DNase"). Longer incubation with DNase led to complete degradation of SMC (data not shown). In both protease deficient strains, SMC was also proteolysed in the presence of DNase, but to a lesser extent compared to wild type cells (Fig. 6, lanes "+ DNase"). These experiments support the notion that ClpX and Lon contribute towards the proteolysis of SMC, and suggest that the presence of DNA protects SMC from proteolytic attack, in a specific or non-specific manner.

### SMC forms rosette-like structures in vitro

**Figure 7:**
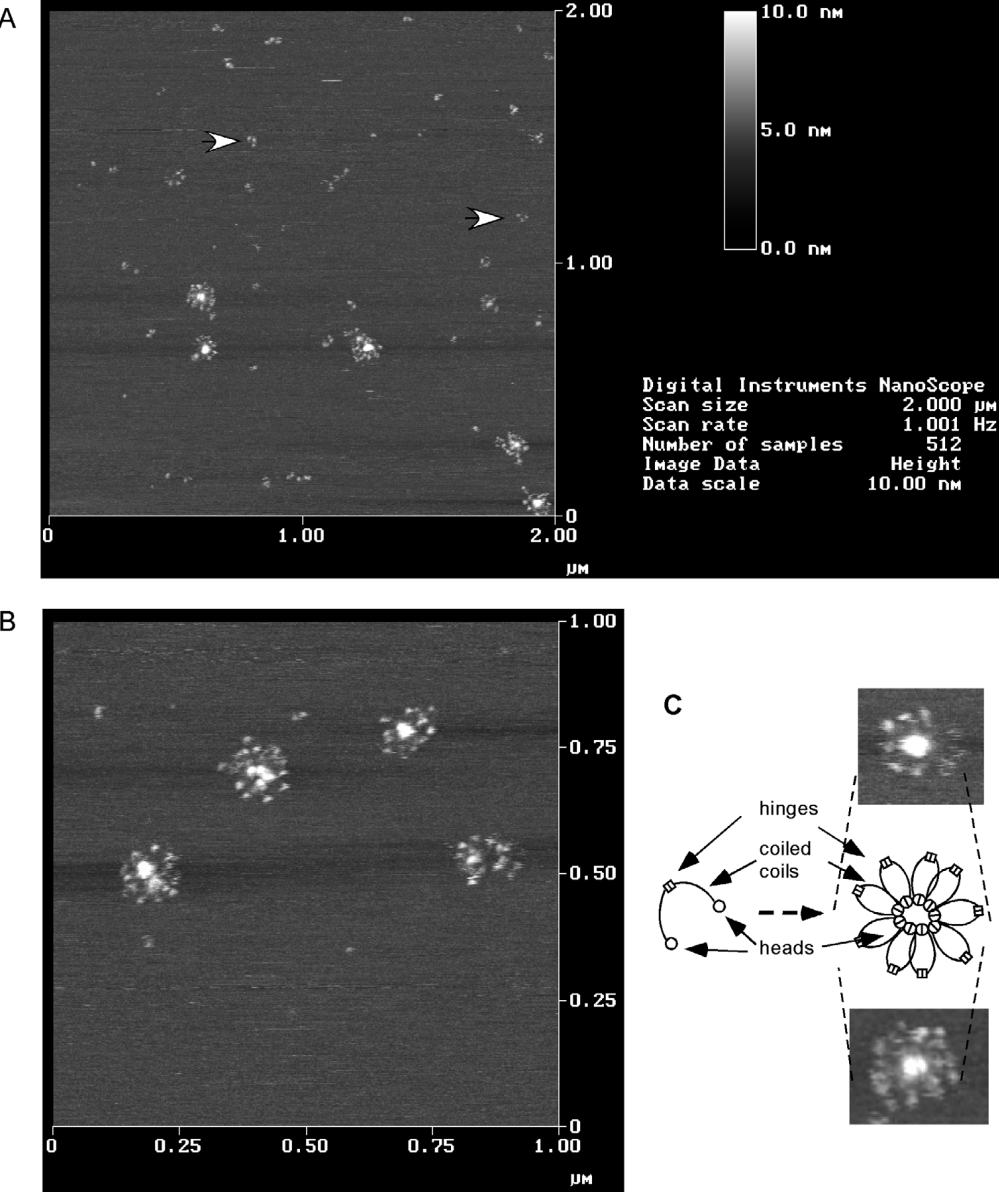
Atomic force microscopical image of SMC on mica in solution. Image shows raw data, just flattened by the software of the instrument. A) Typical field containing single SMC particles (indicated by arrows) and rosette like structures. B) Zoom of another field showing the rosette structures. C) Speculative model of the formation of the rosettes by SMC dimers, in which SMC head domains form the central part of the structures, and arms extend outwards, in which DNA loops could be trapped.

To visualize SMC protein *in vitro*, we employed atomic force microscopy in solution. SMC was mostly present as single particles, whose defined structure was difficult to solve, and frequently, as three globular particles in close proximity (Fig. 7A, indicated by arrows). However, strikingly, we also observed the formation of large regular structures that possess a central core from which thin arms appear to emanate (Fig. 7A and 7B). The outer rim of these sun-like structures showed regularly spaced globular domains. The diameter of the core structure was 40 nm, while the whole structure measured between 110 and 130 nm. Clearly, these super-structures consist of many SMC dimers, and were thus not detectable in gel filtration experiments. To obtain a better idea about the nature of these structures, we analysed purified SMC head domains under the same conditions. Head domains formed globular particles, whose size and height was similar to that of the core structures, but also structures of half or quarter size compared to the 40 nm particles (data not shown). Consistent with this, purified head domains elute as large multimers on gel filtration (data not shown), while the hinge domain forms a dimer only [9]. On the other hand, the structures emanating from the core have an average length of 40 nm, corresponding to the length of 45 to 50 nm for the coiled coil domains [14]. Thus, the data are compatible with the SMC rosette structures having SMC head domains as their core, coiled coil arms emanating outwards, and hinge domains forming the outer rim (Fig. 7C). However, our experiments do not rule out that the core structure consist of hinge domains, while the heads point outwards. SMC rosettes were not found in diluted samples, suggesting that a certain concentration of SMC is required to induce their formation. Few rosettes were detectable for the Walker A mutant SMC, but not for the Walker C mutant protein, suggesting that they are not protein aggregates, but defined structures that require ATPase activity for their efficient assembly. It remains to be investigated if the structures indeed represent the actual architecture of SMC *in vivo*, or if they are purification artefacts. However, the rosette structures are a good basis to explain why SMC forms discrete subcellular structures on the *Bacillus subtilis *chromosomes, and how SMC could condense DNA, e.g. via connection of several DNA loops trapped within coiled coil arms of SMC subunits that appear to emanate from the centres of the structures.

## Discussion

This work provides several new cues about the function and regulation of the prokaryotic SMC complex, showing that its activity depends on a variety of physiological factors and that it is regulated at multiple levels. We have found that ScpA and ScpB are present in a dynamic equilibrium between the free forms and at least two different sub-complexes, which are most likely constituted of a ScpB dimer and a ScpA monomer, and a duplicated form of this complex. *In vitro*, SMC recruited the ScpA/B subcomplex [most likely consisting of 2 ScpA and 4 ScpB (i.e. 2 ScpB dimers)] upon formation of the SMC complex, suggesting that the complex consists of an SMC dimer and of 2 ScpA and 4 ScpB molecules. ScpA has been shown to form mostly monomers, but also dimers in solution, which is supported by the presence of a long coiled coil domain in the central part of ScpA that could mediate dimer formation [7]. ScpB dimers could stabilize such a ScpA dimer. Similar to Scc1 in cohesin [18], a ScpA dimer could bind to SMC with the conserved N-terminal domain binding to one head domain, and the conserved C-terminal domain binding to the other head domain. Alternatively, ScpA could form an antiparallel dimer, presenting two symmetrical and identical binding epitopes, one for each head domain. It was recently shown that ScpA and ScpB reduce the ATPase activity of SMC [23]. If SMC exists in both free and complexed forms *in vivo*, the activity of SMC could be regulated by the ScpAB subcomplex. Indeed, we show that likewise to eukaryotic SMC proteins [39,40], loss of ATPase activity in SMC leads to loss of SMC function *in vivo*, so the equilibrium between ScpAB-bound and free SMC is most likely an important regulatory step within the cell. Interestingly, SMC ATPase mutants were still able to bind to the interacting proteins ScpA and ScpB, albeit with lower affinity. Thus, similar to an mutant ATPase Rad50 protein that still binds to Mre11 [41], but contrarily to the cohesin complex, in which a Walker A mutation in SMC1 (but not in SMC3) abolishes binding to Scc1 [39], assembly of the *B. subtilis *SMC complex does not require ATP binding or hydrolysis activity, although both activities appear to facilitate complex formation. Possibly, opening and closing of the head domains, which apparently requires ATP binding and hydrolysis [42], facilitates binding of ScpA/ScpB, which might constitute a further level of regulation of the activity of the complex.

In the living cell, most of the SMC, ScpA and ScpB molecules are present within defined foci on the nucleoids, one in each cell half, which constitute active chromosome condensation and organization centres [7,9,30]. Thus, the dynamic interaction appears to take place within these bipolar centres. Alternatively, it is also possible that single SMC dimers or complexes are loaded onto newly synthesized DNA at the replication forks (the circular *B. subtilis *chromosome is replicated through the stationary replication machinery located at the middle of the cells [43]), and are translocated to the condensation centres, where they might interact with the SMC complexes present as high order structures at this location. In support of this model, we found that inhibition of DNA replication markedly changed the pattern of localization of the SMC complex. Only centrally located SMC centres were visible under this condition, whereas resumption of replication rapidly restored the bipolar localization of the SMC complex. These experiments reveal the requirement for active replication for the proper positioning of the complex. Possibly, termination of replication stops SMC's condensation activity, coordinating replication/segregation with cell division. In addition to replication, the SMC complex requires proper supercoiling for its assembly in defined condensation centres. Upon inhibition of gyrase, the major source generating negative supercoiling in the cells, the SMC complex was dispersed throughout the cells, and formed subcellular centres in only few cells. Because the SMC complex also failed to localize upon mutation of the SMC Walker A – or C-motifs, we conclude that ATPase activity, ongoing replication and sufficient negative supercoiling are required for proper localization of the SMC complex, revealing that the defined activity of the condensation complex is affected and probably regulated by many physiological and biochemical processes within the cell.

Strikingly, we found high order structures formed by purified SMC *in vitro*, using AFM. The structures appear to consist of rosette-like arrays of SMC dimers that might form through protein-protein interaction of the head domains (hinge domains form dimers only [9]) (Fig. 7). Because SMC binds to DNA by embracing its substrate with the long coiled coil arms, it is possible that many DNA loops are connected through the SMC rosette arrays, which could explain how SMC condenses DNA, and which might be the basis for the formation of the discrete subcellular SMC structures observed *in vivo*. The structures are quite similar to models that were recently put forward to explain the repetitive and cooperative condensation activity of eukaryotic condensin I complex [22], however, further studies need to prove their significance.

An additional level of regulation of the SMC complex takes place based on proteolysis of the SMC protein. While transcription of the *smc *gene is relatively constant at the transition from exponential growth to stationary phase, the SMC protein is rapidly degraded at this point, while only moderate proteolytic turnover of SMC occurs within each cell cycle. Different from exponentially growing cells, SMC was highly prone to proteolysis in cells entering stationary phase, in part mediated by ClpX and LonA proteases. Thus, a switch in its proteolytic susceptibility triggers SMC degradation at the end of exponential growth. It will be interesting to investigate, how this switch is triggered. Interestingly, SMC protein is destabilized in the absence of DNA, indicating that the release of SMC from DNA could lead to rapid degradation of SMC. This is in agreement with the previous finding that the SMC complex is undetectable in cells lacking DNA [7]. Thus, dissociation from DNA could induce proteolytic release of SMC from the rosette structures. If the state of DNA supercoiling is altered as cells enter stationary phase, SMC could loose its ability to bind to DNA and form discrete SMC foci, as shown in our experiments, which might explain the rapid proteolysis of SMC at this growth phase. Data from several laboratories have shown that SMC proteins bind to DNA by embracing their substrate with the long coiled coil arms, and by closing the ring structure through bridging or dimerization of the head domains (HD) [9,20,44]. Head dimerization is apparently mediated by sandwiching of two ATP molecules between HDs, such that the C-motif of one monomer interacts with the ATP bound to the Walker A/B binding pocket on the other monomer, and vice versa [42]. Eventually, ATP is hydrolysed, which will lead to dissociation of HDs, and possibly also to release of DNA. Thus, slow hydrolysis of ATP within the SMC rosette-like superstructure could lead to dissociation and degradation of individual SMC dimers. In analogy, specific degradation of the Scc1 subunit of cohesin leads to dissociation from DNA, and triggers separation of chromosomes during anaphase [45].

In toto, our data suggest the following, highly speculative model for the dynamic regulation of the SMC complex: newly synthesized SMC might bind to DNA at the central replication fork, and introduce loops into the DNA. Binding of the ScpAB subcomplex could stabilize closing of the head domains, and thus ring closure, by reducing ATPase activity, and/or by bridging the head domains in analogy to the cohesin complex. It is unclear, though, when and where ScpA/B bind to SMC. After translocation of the SMC-bound DNA towards the cell poles, SMC might form rosette like structures, possibly one continuous stack of rosettes, or several individual closely spaced rosettes. These structures might connect many individual DNA loops, and could be the basis for the chromosome domain barriers, about 400 of which are known to exist in bacterial chromosomes [46]. Slow ATP hydrolysis might open the ring, leading to dissociation and proteolysis of SMC, such that a new cycle of binding, condensation and release can start. Further experiments will address to resolve remaining questions to understand the detailed mode of action of the SMC complex.

## Methods

### Growth conditions

*E. coli *XL1-Blue (Stratagene) and *B. subtilis *strains were grown in Luria-Bertani (LB) rich medium supplemented with 50 μg/ml ampicillin or other antibiotics where appropriate. All strains used in this study are listed in Table 1. For microscopy, cells were grown in S7_50 _defined medium [47]. For induction of the spac and hyperspank promoters, the culture media were supplemented with 0.1 to 1 mM isopropyl-β-D-thiogalactopyranoside (IPTG).

**Table 1:** strains used in this study

strain	genotype	reference
PY79	wild type	[35]
PGΔ388	*smc::kan*	[35]
JM51	*smc-A *(Walker A mutant) at *amy*	this work
JM52	*smc*-*C *(C-motif mutant) at *amy*	this work
JM59	*smc::kan*, *smc-A *at *amy*	this work
JM60	*smc::kan*, *smc-C *at *amy*	this work
EP58	*smc::kan*, *smc *at *amy*	this work
JM25	*smc-yfp*	[9]
JM8	*scpA-yfp*	[7]
JM57	*dnaA*^ts^, *smc-yfp*	this work
JM45	*dnaA*^ts^, *scpA-yfp*	this work
JM58	*dnaB*^ts^, *smc-yfp*	this work
JM46	*dnaB*^ts^, *scpA-yfp*	this work

### Construction of strains

The strains JM45 (*scpA-yfp, dnaA*^*ts*^), JM46 (*scpA-yfp*, *dnaB*^*ts*^) and JM57 (*smc-yfp, dnaA*^*ts*^) JM58 (*smc-yfp, dnaB*^*ts*^) were constructed by transforming strains KL210 (*dnaA*^*ts*^) and KL58 (*dnaB*^*ts*^) (kind gifts from Catherine Lemon, MIT) with chromosomal DNA from JM8 or JM25 cells (table 1). Mutations K37I (Walker A) and S1090R (C motif) in SMC were created using a PCR-based site-directed mutagenesis kit (QuikChange Site-Directed Mutagenesis Kit, Stratagene). Mutagenesis was performed on a pQE60 plasmid carrying the *smc *gene [9]. The mutant alleles were fully sequenced to verify the mutation. Each mutated *smc *allele was subcloned into plasmid pDR111, in which expression is regulated by induction with IPTG (kind gift of D. Rudner, Harvard Medical School). The resulting plasmids were transformed in *Bacillus subtilis *to yield JM51 (*smc *Walker A mutant at *amy*) and JM52 (C motif SMC mutant at *amy*) (table 1). In order to see the phenotype of the *smc *mutants, the original *smc *gene was disrupted by transforming the respective mutant with chromosomal DNA from PGΔ388, generating strains JM59 (*smc *walker A mutant at *amy*, *smc::kan*) and JM60 (*smc *C motif mutant at *amy*, *smc::kan*), which express solely *smc *mutant alleles from the *amy *locus, as verified using Western blotting (data not shown). EP58 was constructed by transformation of a strain carrying the *smc *gene at the *amy *locus (kind gift from Eduardo Gonzales-Pastor, Centro de Astrobiología, Madrid, Spain) with chromosomal DNA from PGΔ388. The *clpX *and *lonA *mutant strains are described in [38].

### Primer extension studies

Total RNA from cells grown at various growth stages was isolated using the Qiagen RNeasy mini kit. A primer complementary to the 5' end of the *smc *mRNA was labelled with [^32^P]-γ-ATP using PNK, and was used in the primer extension reaction with reverse transcriptase (Super ScriptII) enzyme. The reaction products were separated on a 6% urea-polyacrylamide gel next to a sequencing reaction carried out using the same primer and a plasmid as template that carried the DNA region coding for the *rncS/smc/ftsY *region.

### Preparation of protein extracts

For the preparation of protein extracts from cell lysates, culture volumes corresponding to equal OD_600 *nm *_values of 5.0 were harvested from growing PY79 cells at different phases. This procedure ensured that similar amounts of cells were withdrawn for protein extract preparation when cells of different growth stages had to be compared. The isolated cell pellets were lysed by sonication. After determination of protein concentration, cell extracts were loaded onto SDS-PAGE, and equal protein levels between the lanes were visually confirmed by Coomassie staining. Western blot analyses were performed using antibodies specific for SMC.

### Construction of His-tagged SMC, ScpA, and ScpB, and purification of proteins

Proteins were purified using a two step column protocol as described in [9]. Antibodies against an internal fragment of SMC or against whole-ScpB were raised in rabbits (Eurogentec), and reacted with a single band corresponding to the respective protein in Western blot experiments.

### Analytical gel filtration and sucrose gradient centrifugation

Gel filtration was performed on Superdex 200 10/300 GL column from Amersham-Pharmacia Biotech. Proteins were mixed to give a final volume of 500 μl in HEPES A buffer (50 mM HEPES, 300 mM NaCl, pH 8.0), and were incubated for 30 min before applying to the column. For gradient centrifugation, 5 to 20% sucrose gradients were spun at 165,000 × *g *for 15 h. 1 ml fractions were withdrawn and subjected to SDS-PAGE analysis. Standard proteins used were thyroglobulin (670 kDa), γ-globulin (158 kDa), bovine serum albumin (66 kDa), ovalbumin (44 kDa), chymotrypsinogen (25 kDa), myoglobin (17 kDa), cytochrome C (14 kDa), and aprotinin (6.5 kDa).

### Atomic force microscopy

Imaging was carried out in buffer solution using a Multimode Nanoscope IIIa (Digital Instruments, Santa Barbara, CA) working in tapping mode with a fluid cell without o-ring seal. The cantilevers (OLYMPUS, OMCL TR800PSA, 100 μm, 0.68 N/m) were operated at an excitation frequency around 19 kHz. 10 μl of protein in solution were allowed to adsorb on freshly cleaved mica at a final concentration of about 0.1 μg/μl in buffer (50 Hepes, pH 8.0, 300 NaCl) for 30 min. Afterwards the sample was gently rinsed with the same buffer to remove excess protein. It was kept under buffer solution until scanning.

### Image acquisition

Fluorescence microscopy was performed as described in [7]. DNA was stained with 4',6-diamidino-2-phenylindole (DAPI; final concentration 0.2 ng/ml) and membranes were stained with FM4-64 (final concentration 1 nM).

## Authors' contributions

J M performed the experiments shown in Figs. 4, 5, 6, A V performed the biochemical experiments shown in Figs. 1, 2, 3 (except for Fig. 1H), C R purified the SMC head domains and performed the experiment shown in Fig. 1H, J S performed the AFM experiments shown in Fig. 7, R G helped with the design of the AFM experiments, PLG performed the experiment shown in Fig. 4F, conceived the study, and participated in its design and coordination. All authors read and approved the final manuscript.
